# Supplementary material for: Pharmacist-Driven Alcohol Use Disorder Screening May Increase Inpatient Utilization of Extended-Release Naltrexone: A Single Center Pilot Study
Source: Pharmacy (Basel). 2024 Feb 1;12(1):26. doi: 10.3390/pharmacy12010026 (PMC10892525; doi:10.3390/pharmacy12010026)
Supplement: Supplementary file 1 [file pharmacy-12-00026-s001.zip › pharmacy-2769466-supplementary.pdf]

## Pharmacist-Driven Extended-Release Naltrexone Screening Tool

**Extended-Release Naltrexone (XR-NTX) Screening Tool**

HPI:

Allergies: Ht/ Wt:

Pregnancy/Lactation Status:

Urine Drug Screen:

| | | |
|---|---|---|
| ☐ Methamphetamine/ Amphetamine | ☐ Benzodiazepine | ☐ Phencyclidine |
| ☐ MDMA | ☐ Cocaine | ☐ Barbiturate |
| ☐ Methadone | ☐ Buprenorphine | ☐ Opiates |
| ☐ THC | ☐ TCA | ☐ Ethyl Alcohol:_________ |

**Based on the following criteria, the patient is a candidate for XR-NTX (Vivitrol®).**

- ☐ Diagnosis of Alcohol Use Disorder
  → Amount of alcohol consumed:
- ☐ Urine drug screens negative for opioids – DATE: _________
- ☐ History of tolerability to oral agents related to XR-NTX
- ☐ History of tolerability to XR-NTX

**Based on the following criteria, the patient is NOT candidate for XR-NTX (Vivitrol®).**

- ☐ Actively on opioid treatment or withdrawing from opioids
- ☐ Opioid use within the past 7-10 days
- ☐ Pregnant
- ☐ Severe renal impairment
- ☐ AST/ALT ≥ 3x upper limit

*Meets XR-NTX Criteria:* Based on the above criteria, and the patient's substance abuse history, please consider the following to aid in medication adherence and increase time to first heavy drinking day:
*Medication regimen…*

*Does not meet XR-NTX Criteria:* Based on the above criteria, patient is not a candidate to receive Vivitrol at this time. Pharmacist will continue to monitor medications.

*Use of this screening tool is restricted to those with express permission from the authors. Use must be cited appropriately.
